# Enhanced photocurrent production by bio-dyes of photosynthetic macromolecules on designed TiO_2_ film

**DOI:** 10.1038/srep09375

**Published:** 2015-03-20

**Authors:** Daoyong Yu, Mengfei Wang, Guoliang Zhu, Baosheng Ge, Shuang Liu, Fang Huang

**Affiliations:** 1 State Key Laboratory of Heavy Oil Processing, China University of Petroleum (East China), Qingdao 266580, China; 2 Center for Bioengineering and Biotechnology, China University of Petroleum (East China), Qingdao 266580, China

## Abstract

The macromolecular pigment-protein complex has the merit of high efficiency for light-energy capture and transfer after long-term photosynthetic evolution. Here bio-dyes of *A.* *platensis* photosystem I (PSI) and spinach light-harvesting complex II (LHCII) are spontaneously sensitized on three types of designed TiO_2_ films, to assess the effects of pigment-protein complex on the performance of bio-dye sensitized solar cells (SSC). Adsorption models of bio-dyes are proposed based on the 3D structures of PSI and LHCII, and the size of particles and inner pores in the TiO_2_ film. PSI shows its merit of high efficiency for captured energy transfer, charge separation and transfer in the electron transfer chain (ETC), and electron injection from F_B_ to the TiO_2_ conducting band. After optimization, the best short current (J_SC_) and photoelectric conversion efficiency (η) of PSI-SSC and LHCII-SSC are 1.31 mA cm^-2^ and 0.47%, and 1.51 mA cm^-2^ and 0.52%, respectively. The potential for further improvement of this PSI based SSC is significant and could lead to better utilization of solar energy.

It was shown in 1991 that the use of mesoporous TiO_2_ film and an efficient charge injection dye raised the dye-sensitized solar cell (DSSC) efficiency from less than 1% to more than 7%^1^. Since then DSSC has attracted great attention and is very promising due to its low-cost and flexible fabrication^2^,^3^. To date, the highest efficiency record of over 12% for prototype DSSC is still held by TiO_2_ film in association with optimal device structure^4^,^5^. The performance of DSSC is mainly based on the dye sensitizer, which acts as an electron pump to transfer the sunlight energy into the electronic potential. Natural photo-sensitizers have become a viable alternative to expensive and rare organic sensitizers because of their low cost, the abundance of raw materials with no associated environmental threat^6^. Intensive research efforts have been directed toward the application of several highly efficient light-harvesting photosynthetic pigment-protein complexes, including reaction centers, photosystem I (PSI), and photosystem II, as key components in the light-triggered generation of fuels or electrical power^7^,^8^. An algae light-harvesting antenna, phycobilisome based solar cell had been fabricated by assembly on ZnO nanowires^9^ and TiO_2_^10^, and this shows that phycobilisome sensitization coupling with Chlorin e_6_ can expand the absorption spectrum range, increase the short-circuit current (J_sc_) and improve the photoelectric conversion efficiency (η), all of which were higher than the sum of phycobilisome and Chlorin e_6_ sensitization alone.

PSI is associated with electron transport and exists as a large, multi-subunit complex with dozens of transmembrane spanning domains. Cyanobacteria PSI precisely orchestrates 127 cofactors, which contain 96 chlorophylls, 22 carotenoids, 2 phylloquinones, 3 Fe_4_-S_4_ clusters and 4 lipids^11^, achieving efficient quantum coherent energy transfer^12^ and an unprecedented quantum yield of nearly 1.0^13^. This high quantum yield can not be achieved by any man-made photoelectronic device and has led to PSI being studied as a candidate for many applications^14^,^15^,^16^,^17^,^18^. Research on PSI biophotovoltaics had focused on proof-of-principle devices that study immobilized PSI complexes and isolated reaction centers in self-assembled monolayers on flat electrodes^19^,^20^,^21^,^22^, and the electrical power output of these biophotovoltaics has been very low. PSI of thermophilic cyanobacteria was reconstituted on quinone-monolayer-modified electrodes with dichloroindophenol (DCIP)/ascorbate as sacrificial electron donor^23^,^24^. A multi-layered PSI was assembled on electrodes, and data were collected at an overpotential of +0.1 V (approximately +300 mV vs. Ag/AgCl)^25^. An alternative approach to form integrated PSI assemblies on electrodes has been introduced, in which PSI with the associated Pt nanoclusters (NP) was modified with thioaniline and electropolymerized with thioaniline functionalized Pt NP to yield a bis-aniline crosslinked PSI/Pt NP composite for the generation of photocurrents with dichlorophenol indophenol (DCPIP)/ascorbate as sacrificial electron donor^26^. Isolated PSI was immobilised on a gold electrode surface via an Os complex containing redox polymer hydrogel which act as both immobilization matrix and electron donor for PSI, and a catalytic photocurrent was observed upon illumination with addition of methyl viologen as sacrificial electron acceptor^27^. Recently, an integrated PSI/PSII assembly on electrodes showed directional generation of photocurrents^28^, which allowed the exclusion of the sacrificial electron donor (DCPIP/ascorbate) and the use of water electrolyte donor for scavenging P_680_^+^^28^. In all of these systems, the generation of photocurrents has involved the use of sacrificial electron donor or acceptor, or the biasing of electrode at a potential capable of reducing the photogenerated holes.

A recent promising application of isolated PSI is its integration in biohybrid DSSC^29^,^30^. PSI stabilized by surfactant peptides functioned as both the light-harvester and charge separator self-assembled on nanostructured TiO_2_ or ZnO electrodes using Co(II/III)-tris-bipyridine as electron transfer mediator, and achieving the best J_sc_ 362 μA cm^−2^ and η 0.08% for a PSI-TiO_2_ solar cell^29^. A robust red algal PSI associated with its light harvesting antenna (LHCI) coupled with nanocrystalline n-type semiconductors of TiO_2_ and α-Fe_2_O_3_ as biophotoanodes using electrolyte (I^−^/I_3_^−^) as electron transfer mediator, has also achieved the best J_sc_ 56.9 μA cm^−2^ and η 0.17% for PSI-LHCI/α-Fe_2_O_3_ solar cell^30^.

Light-harvesting complex II (LHCII) is the light-harvesting antenna of photosynthetic bacteria^31^ and plants^32^ with high absorption coefficient, wide spectrum range, high quantum conversion efficiency and environmental compatibility. Spinach LHCII, the most abundant integral membrane protein in chloroplasts, exists as trimer and binds about half of the thylakoid chlorophyll molecules^32^, and can be adsorbed on TiO_2_ by carboxyl anchoring groups located at the stromal side^33^. Therefore, LHCII could be a more promising sensitizer than some other photosynthetic complexes. When LHCII was first immobilized on a TiO_2_ nanostructured film it was found that it can act as an effective photosensitizer with a wide absorption range and is a promising candidate for photoenergy conversion materials^33^. The effect of adsorbance and light intensity on the photovoltaic performance of LHCII/TiO_2_ solar cells was also investigated^34^ and it was found that LHCII can be stably assembled on the surface layers of TiO_2_ film without apparent pigment disassembling. Adsorbance is an important factor affecting the cell performance and for next generation of LHCII-sensitized solar cells (LHCII-SSC), the size of the TiO_2_ particles and the thickness and porous structure of the TiO_2_ films should be optimized to gain an optimal three-dimensional adsorption^34^. Recently, a possible sensitization model of a chlorophyll-chlorophyll charge transfer state has been proposed for effective coupling with the TiO_2_ surface and thus injecting electrons into the TiO_2_ conduction band^35^, and bio-dyes such as pigment-protein complexes of purple bacteria have been verified to be promising natural sensitizer candidates for fabricating visible-NIR responsive DSSC^36^.

In this work, we designed three types of TiO_2_ films on FTO glass as TiO_2_ electrode for the sensitization of *A. platensis* PSI and spinach LHCII, to explore the effects of pigment-protein complex on the performance of biohybrid photovoltaic cells. This involves consideration of the effects of the TiO_2_ morphology on the attachment and performance of PSI and LHCII on its surface and a comparison of the two materials.

## Results

The adsorption capacity and saturation time are probably determined by the size of sensitizers and the porous structure of the TiO_2_ film, and for LHCII-SSC, the size of TiO_2_ particles, the thickness, and the porous structure of the TiO_2_ film should be optimized to gain an optimal three-dimensional adsorption^34^. Here, we implement two photosynthetic protein complexes, *A.* *platensis* PSI and spinach LHCII, as bio-sensitizer to assemble the bio-photovoltaics. The trimeric PSI forms a clover-leaf-structure with a diameter of 22 nm^37^, and the monomeric PSI and trimeric LHCII show maximal diameters of ~13 nm and ~6 nm respectively as measured by PyMOL (Figure S1).

### Design of TiO_2_ Electrodes

The basic design principle for solar cells is to increase the optical absorption of the active layer and/or reduce the electron loss during transport, and the tailoring of materials to optimize light harvesting and electron transport is an effective means of creating high performance solar cells^38^.

In traditional DSSCs, a TiO_2_ electrode film should be as thick as possible so as to adsorb enough dye molecules and thus achieve sufficient absorption of incident light. However, the practically feasible thickness for a TiO_2_ electrode film is limited by the diffusion length of the electrons in the nanocrystalline TiO_2_ film. TiO_2_ nanoparticles are employed to provide a large internal surface area for dye adsorption, but this simultaneously gives a large TiO_2_/electrolyte interface, which increases the probability for charge recombination and therefore acts to shorten the diffusion length of the electrons. The optimal thickness for the DSSC photoelectrode film is typically around 15 μm. However, the optical absorption of a dye-sensitized nanocrystalline TiO_2_ film with this thickness has been shown to be incomplete, i.e., there is a loss of incident light due to partial transmission. In traditional DSSCs, this problem is partially circumvented by including large sized particles in the nanocrystalline film to scatter the light and thus extend the light pathway within the photoelectrode film^38^. By the addition of a scattering layer of large TiO_2_ particles on the transparent nanocrystalline TiO_2_ film, the light introduced from the substrate side can be reflected back to the transparent TiO_2_ film. In this way, the traveling distance of the incident light is doubled so that the optical absorption of the photoelectrode can be effectively improved.

According to the above principle and the geometric dimensions of *A.* *platensis* PSI and spinach LHCII, three types of TiO_2_ electrodes were designed and fabricated by layer-by-layer screen printing^39^, and each layer was ~2 μm thick. The surface morphology of the TiO_2_ films, including the size of TiO_2_ particles and the porous structure, was characterized by scanning electron microscopy (SEM).

**Figure 1:**
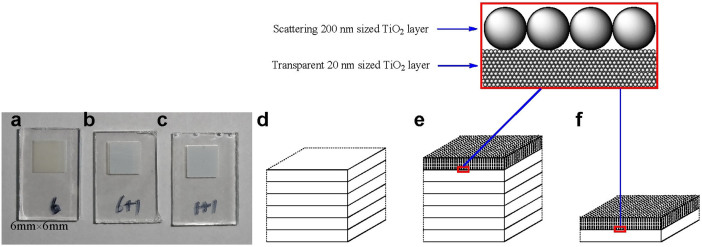
Top view photos of TiO_2_ electrodes of (a) 6 type, (b) 6+1 type, and (c) 1+1 type, and schematic presentation of electrodes of (d) 6 type, (e) 6+1 type, and (f) 1+1 type.

**Figure 2:**
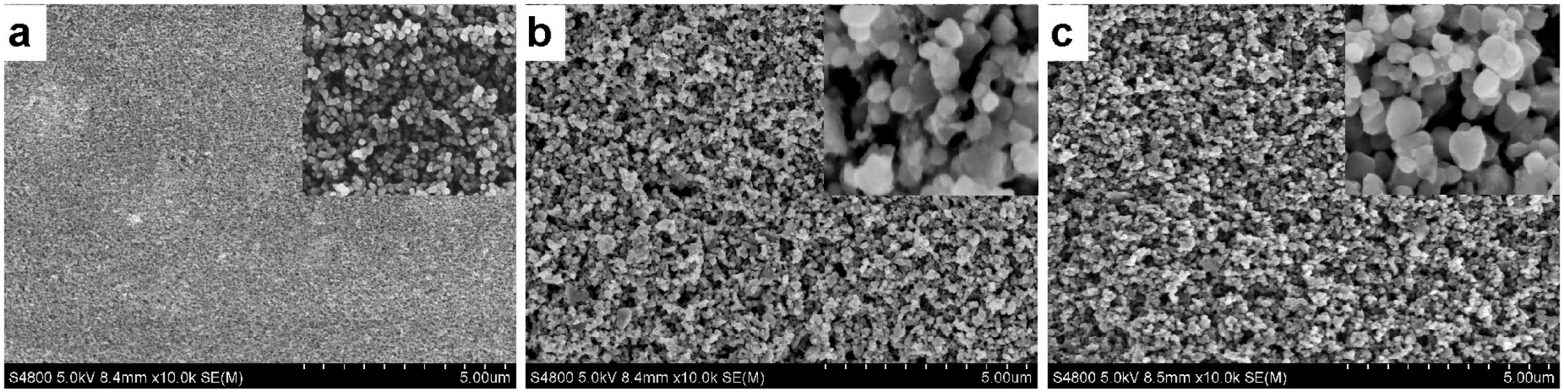
Top view SEM images of nanocrystalline TiO_2_ electrodes. (a) 6 type; (b) 6+1 type; (c) 1+1 type. Inner images' width 1 μm.

1. 6 type TiO_2_ electrode (Figure 1a and d) which was composed of 6 layers of ~2 μm thick transparent layer of mesoporous TiO_2_ with ~20 nm particle size (Figure 2a). This provides a large surface area for traditional sensitizer adsorption and good electron transport to the FTO substrate.
2. 6+1 type TiO_2_ electrode (Figure 1b and e) which was composed of 6 layers of ~2 μm thick transparent layer of mesoporous TiO_2_ with ~20 nm particle size, and a light scattering layer on top of the mesoporous film, consisting of a ~2 µm porous layer containing ~200 nm sized TiO_2_ particles (Figure 2b).
3. 1+1 type TiO_2_ electrode (Figure 1c and f) which was composed of one layer of ~2 μm thick transparent layer of mesoporous TiO_2_ with ~20 nm particle size, and a light scattering layer on the top of the mesoporous film, consisting of a ~2 µm porous layer containing ~200 nm sized TiO_2_ particles (Figure 2c).

### Adsorption of PSI and LHCII on TiO_2_ Electrodes

The dye should have anchoring groups (-COOH, -H_2_PO_3_, -SO_3_H, etc.) to bind it strongly onto the semiconductor surface, and the standard anchoring group for sensitizers is carboxylic acid (-COOH) which reacts with surface hydroxyl groups to form chemical bonds^3^. Thus, it is likely that assembly of PSI or LHCII on the electrode is facilitated by the many carboxyl residues such as aspartic (D) or glutamic acid (E), in the protein sequences of PSI or LHCII, which bind easily to the surface of TiO_2_. Possible binding modes of a -COOH group to TiO_2_ include coordinate covalent, carboxylate, and hydrogen bonds^40^.

For PSI monomer, there are 102 carboxyl residues (55E+47D) at the stromal side, and there are 58 carboxyl residues (15E+43D) at the lumenal side. The stromal side has more carboxyl groups (Figure S2) on the surface and tends to be preferentially fixed on the TiO_2_ surface. Similarly for LHCII monomer, there are 18 carboxyl residues (10E+8D) at the stromal side, and there are only 7 carboxyl residues (4E+3D) at the lumenal side. The stromal side has more carboxyl groups (Figure S3) on the surface, and will tend to be preferentially fixed on TiO_2_ surface. Thus, for both PSI trimer and LHCII trimer, the stromal side surface with more carboxy groups (Figure S4) will tends to be preferentially fixed on the TiO_2_ surface.

**Figure 3:**
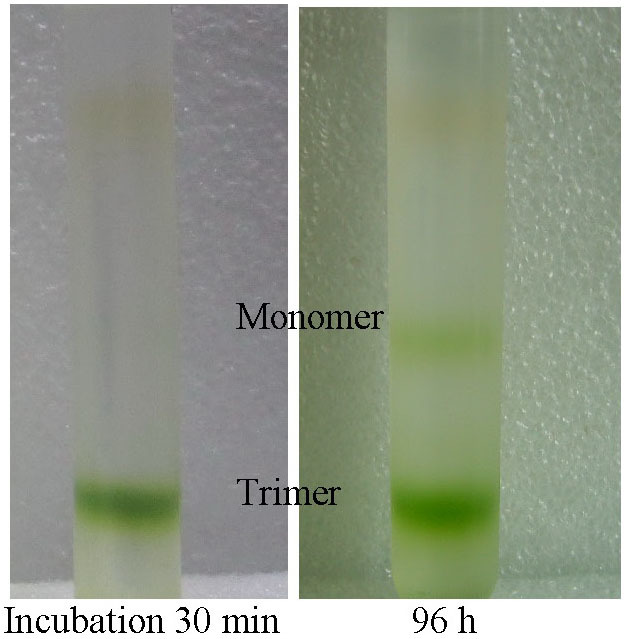
Sucrose density gradient centrifugation of DDM-solubilized PSI after incubation of 30 min and 96 h respectively, performed by the method described in Ref. 42.

**Figure 4:**
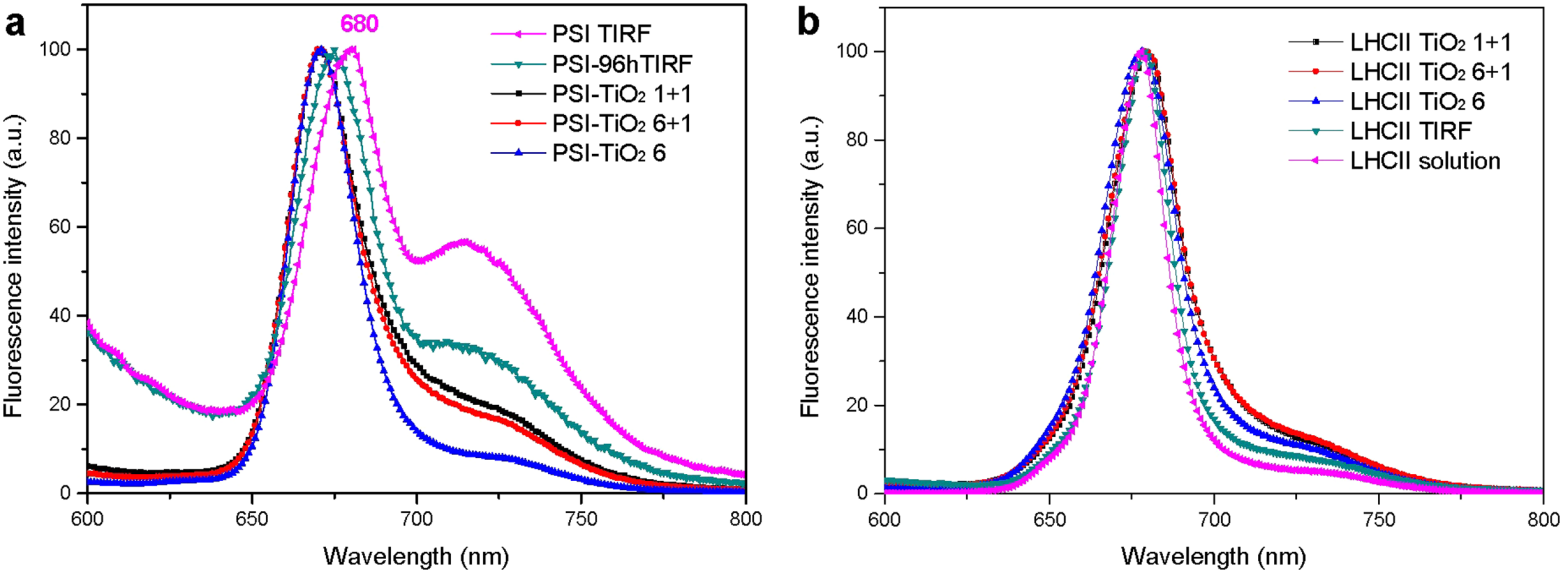
Fluorescence spectra of (a) PSI solutions and PSI-TiO_2_ electrodes, (b) LHCII solutions and LHCII-TiO_2_ electrodes, normalized at maxima.

Here surfactant *n*-Dodecyl-β-D-maltoside (DDM) instead of Triton X-100 was used to keep *A.* *platensis* PSI or Spinach LHCII suspended in solution, and to inhibit the formation of multilayer on the TiO_2_ surface^41^. DDM solubilized PSI and LHCII mainly exist as trimers^34^,^42^, and the absorption spectra of PSI and LHCII measured at room temperature are presented in Figure S5. PSI trimer solubilized in DDM solution is not very stable and some trimers dissociate to monomers in 96 hours (Figure 3). The TIRF spectrum of PSI solution changed drastically after 96 hours incubation (Figure 4a and Figure S6a). The peak at ~720 nm decreased significantly, in accordance with some dissociation of PSI from trimeric to monomeric form because of the interaction with DDM. The fluorescence spectra of PSI-TiO_2_ electrodes are similar to the TIRF spectrum of PSI-96h, indicating that some of PSI is fixed on TiO_2_ as monomers. In addition, it was found that at the high concentration of equivalent 400 μg Chl/mL, the stability of PSI trimer is much better than that of PSI at a concentration of equivalent 80 μg Chl/mL (Figure S6b).

Computations revealed similar results for the bulk properties of excitation migration, such as quantum yield and average excitation lifetime, for monomeric and trimeric forms of PSI^43^. PSI from *Thermosynechococcus* elongatus was found to be similarly photoactive between the trimeric native form and the monomer isolated by disassembling the trimer into its constituent monomers. The similar photoactivity between monomer and trimer indicates that one can use either form^44^.

Spinach LHCII trimer solubilized in DDM solution is stable for more than two weeks^34^. The fluorescence spectra of LHCII-TiO_2_ electrodes are similar to the TIRF spectrum of LHCII solution (Figure 4b), indicating that LHCII is fixed on TiO_2_ as the intact trimeric form. Compared with the emission of LHCII trimer in solution, the enhanced far-red emission from 690 to 750 nm indicates there is some trimer-trimer interaction between the LHCII trimers on the surface of TiO_2_ and TIRF quartz glass^45^.

PSI and LHCII are macromolecules, and the size screening effect of the mesopore in TiO_2_ layer controls the adsorption depth of these macromolecular bio-dyes. It is diffusion limited process for their adsorption in the thick TiO_2_ film, and a long time is therefore required for equilibration. Most of the adsorption by covalent interaction of carboxyl groups with TiO_2_ surface is irreversible, and it is impossible to desorb these macromolecules completely off TiO_2_. On the other hand, pigments of PSI and LHCII are noncovalent binding, and can be easily extracted by a mixed solvent of 80% acetone/water. After extraction, nearly all pigments were removed from the sensitized TiO_2_ electrodes as verified by their fluorescence spectra (Figure S7 and S8). Therefore, the adsorbance of LHCII and PSI on TiO_2_ electrodes was measured by fluorescence spectrometry (Figure S9 and S10) in units of equivalent chlorophylls (Table 1).

**Figure 5:**
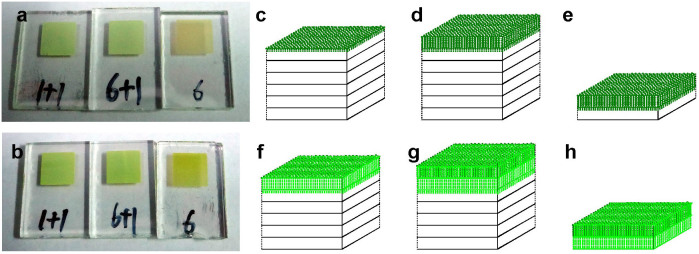
(a) Top view photos of PSI-sensitized TiO_2_ electrodes of 1+1, 6+1 and 6 types. (b) Top view photos of LHCII-sensitized TiO_2_ electrodes of 1+1, 6+1 and 6 types. Adsorption models of PSI@TiO_2_ electrodes of (c) 6, (d) 6+1, and (e) 1+1 types. Adsorption models of LHCII@TiO_2_ electrodes of (f) 6, (g) 6+1, and (h) 1+1 types.

Comparing the 6+1 with the 6 type electrode, addition of the scattering layer helps to increase the adsorbance of PSI (Table 1). The adsorbances of PSI on the 6+1 and 1+1 type electrodes are similar, which indicates that enhancing the thickness of the transparent TiO_2_ film cannot increase the adsorbance of PSI (Table 1). The diameters of PSI monomer and trimer are ~13 nm and ~22 nm respectively, which are smaller than the mesopore size in the 200 nm TiO_2_ scattering layer, and larger than the mesopore size in the 20 nm TiO_2_ transparent layer (Figure 2). Thus it is reasonable to deduce that PSI can be fixed not only on the surface but also in the inner mesopore of the scattering layer, where it can reach the surface of transparent TiO_2_ layer, but cannot diffuse into the inner deeper mesopore of the transparent TiO_2_ layer. A schematic adsorption model of PSI on TiO_2_ electrode is shown in Figure 5. There is only a shallow surface adsorption of PSI on the transparent layer of 6 type electrode (Figure 5c), but there is a more complete inner adsorption of PSI on the scattering layer of both 6+1 and 1+1 type electrodes (Figure 5d, e).

For a 150–300 nm thin TiO_2_ layer^35^, the calculated adsorbance for a fully covered LHCII monolayer is only ~0.2 μg Chl cm^−2^ with enhanced LHCII immobilization via electrostatic interaction with amine-functionalized photoanodes. However, for a thick TiO_2_ layer of several μm, the adsorbance is much higher reaching 4.7–6.0 μg Chl cm^−2^ (Table 1). Comparing the 6+1 type with the 6 type electrode, addition of the scattering layer helps to increase the adsorbance of LHCII (Table 1). But the adsorbance of LHCII on the 6+1 type electrode is larger than that on the 1+1 type electrode, which indicates that enhancing the thickness of the transparent layer can increase the adsorbance of LHCII. The diameter of LHCII trimer is ~6 nm, which is smaller than the pore size in the 200 nm TiO_2_ scattering layer, and in the 20 nm TiO_2_ transparent layer (Figure 2). Thus it is reasonable to deduce that LHCII can be fixed not only on the surface but also in the inner mesopores of both the scattering layer and the transparent TiO_2_ layer (Figure 5), and the depth of LHCII adsorption in the transparent layer is larger than 2 μm (Figure 5f, g).

### Characterization of PSI and LHCII Sensitized Solar Cells (SSC)

In order to explore the relationship between bio-solar cell performance and the bio-dye type, and the TiO_2_ film structure, solar cells sensitized with PSI or LHCII on the three types of TiO_2_ electrode were assembled and characterized.

**Figure 6:**
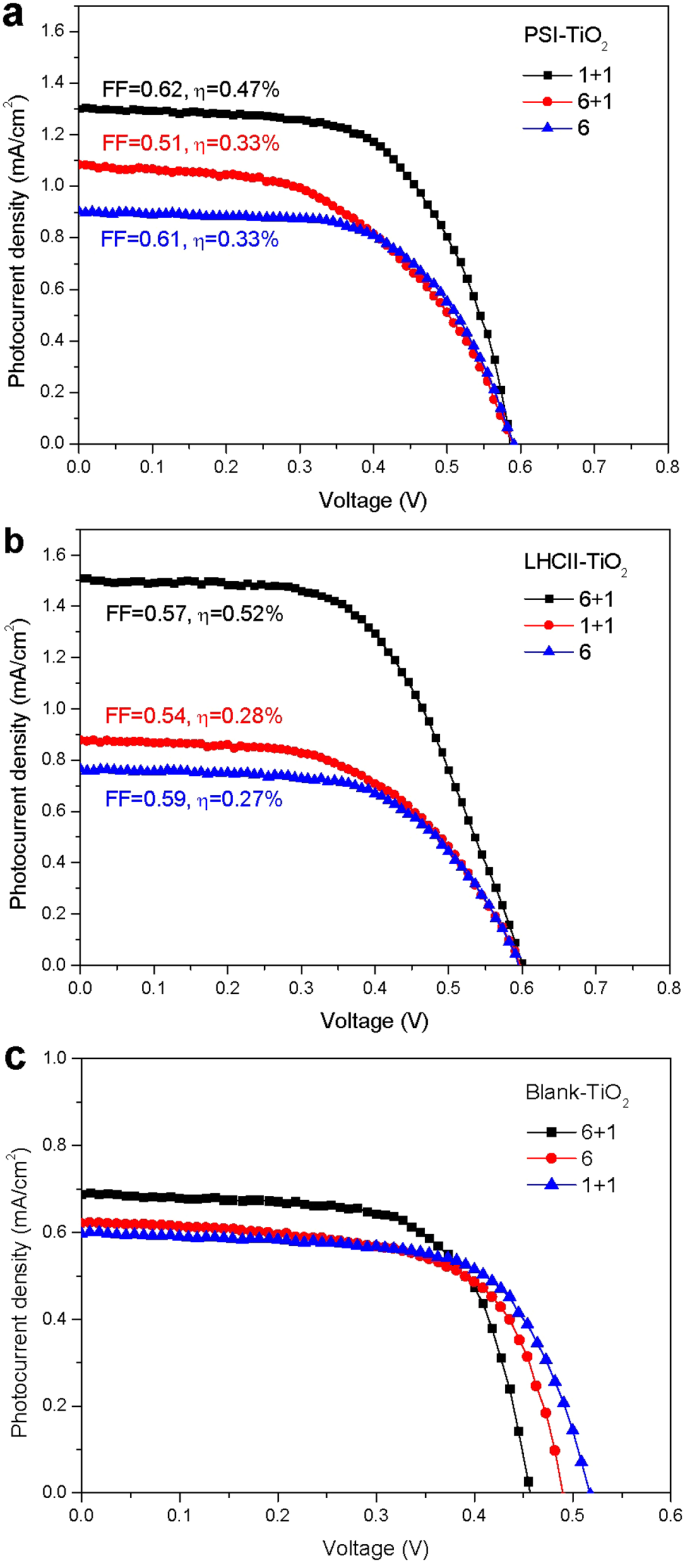
The current-voltage characteristics measured under AM 1.5 G solar irradiance (100 mW cm^−2^ photon flux) for (a) PSI-sensitized, (b) LHCII-sensitized, and (c) blank solar cells with different TiO_2_ electrodes.

Light harvesting, i.e. photon capture, is closely related to the adsorbance of sensitizer. Generally, both the short-circuit current J_SC_ and efficiency η increase with adsorbance^34^. The adsorbances of bio-dye on the three types of electrodes are different from each other, and both J_SC_ and η tend to increase with adsorbance (Figure 6a, b), which is higher than those of the blank solar cells (Figure 6c). Thus the adsorbance of bio-dye has an important effect on the performance of the sensitized solar cell (SSC).

Other important factors influencing the SSC are the series and shunt resistances of the equivalent circuit for SSC (Figure S11). During operation, the efficiency of solar cells is reduced by the dissipation of power across internal resistances. These parasitic resistances can be modeled as a parallel shunt resistance (R_SH_) and series resistance (R_S_). For an ideal cell, R_SH_ should be infinite and should not provide an alternate path for current to flow, while R_S_ should be zero, resulting in no further voltage drop before the load. Decreasing R_SH_ and increasing R_S_ will decrease the fill factor (FF) and P_MAX_. If R_SH_ is decreased too much, V_OC_ will drop, while increasing R_S_ excessively can cause J_SC_ to drop.

R_S_ is closely related to the structure of the TiO_2_ electrode, the thicker the film of TiO_2_, the greater the internal R_S_ of SSC. For PSI-SSC, the adsorbances of PSI on the 6+1 and 1+1 type electrodes are similar, but R_S6+1_>R_S1+1_, with the result that the efficiency of 1+1 type PSI-SSC is better than that of 6+1 type PSI-SSC. Compared with the 6 type TiO_2_ electrode, the adsorbance of 6+1 type is greater than that of the 6 type, but the additional adsorbance is mainly distributed in the scattering layer. The electrons generated in the scattering layer need to pass a long distance to the collecting FTO film, and R_S_ increases. These combinations result in a similar efficiency for 6 and 6+1 type PSI-SSC.

For LHCII-SSC, the adsorption of LHCII in the transparent TiO_2_ layer is much deeper, and the addition of the scattering layer increases the light absorption by LHCII in the transparent layer and also increases the adsorbance of LHCII. These result in the J_sc_ and η of 6+1 type LHCII-SSC being nearly double that of 6 type LHCII-SSC.

**Figure 7:**
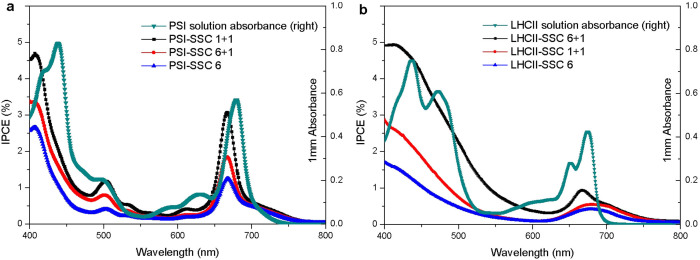
Incident photocurrent conversion efficiencies (IPCE) as a function of wavelength. (a) PSI-SSC, (b) LHCII-SSC with photoanodes possessing different layers of TiO_2_ on conductive FTO glass.

The incident monochromatic photon-to-electric current conversion efficiency (IPCE) as a function of wavelength is presented in Figure 7. The IPCE spectra of PSI-SSC closely follow the absorption spectrum of PSI in solution (Figure 7a), which means that efficiencies of photocurrent generated by the captured photons with different energies are similar. This verifies that any captured photon with different energy can cause charge separation in P_700_ and transfer in ETC and the final electron injection from F_B_ to the TiO_2_ conducting band with nearly identical efficiency. DDM solubilized trimeric PSI is not very stable and some trimers dissociate to monomers (Figure 3), and these small monomers are preferred to be adsorbed in the deeper of TiO_2_ film. Thus the IPCE spectra of PSI-SSC should be similar to the absorption spectrum of monomeric PSI in solution, which is theoretically narrower and with some blue shift compared with that of trimeric PSI.

The IPCE spectra of LHCII-SSC is similar to the absorption spectrum of LHCII in solution (Figure 7b), indicating that LHCII can be excited by the captured photons and inject electrons into the conducting band of the TiO_2_ film. Compared with the absorption spectrum of LHCII in solution, the IPCE spectra of LHCII-SSC is relatively higher in the range of 400–500 nm, possibly indicating that there is a differential injection efficiency of the LHCII electrons excited by the captured photons with different energy, the higher the photon energy, the higher the injection efficiency.

### Comparison of PSI-SSC and LHCII-SSC

The adsorbance of sensitizer and the structure of TiO_2_ film are two main factors that affect the performance of DSSC, but now the effect of the bio-dye type on the cell performance is considered.

**Figure 8:**
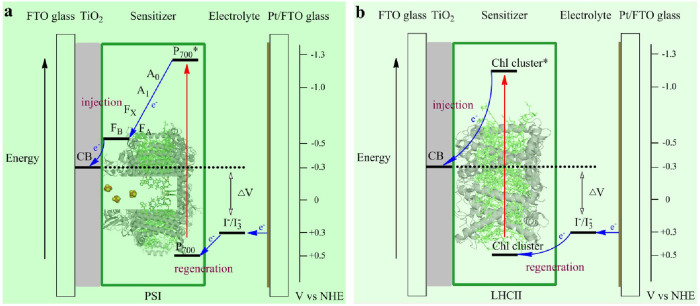
Simple energy level diagram for bio-sensitized solar cell. The potentials are shown for (a) PSI-SSC and (b) LHCII-SSC. The primary free-energy losses are associated with electron injection from the excited sensitizer into the TiO_2_ conduction band (CB) and regeneration of the dye by the redox couple.

As shown in Figure 6, the open circuit voltage of PSI-SSC and LHCII-SSC is 0.59 and 0.60 V respectively, which is approximately indicated by the splitting between the TiO_2_ Fermi level and the chemical potential of the redox electrolyte (Figure 8). The highly directional and selective adsorption of the macromolecular bio-sensitizers is predominantly facilitated by their abundant carboxyl groups at the surface of the stromal side.

In vivo, the task of PSI is to supply the cell with strong reducing potential (electron). PSI uses light energy to transport electrons from reduced plastocyanin or cytochrome c_6_ to soluble ferredoxin, generating chemical species with a redox potential low enough ultimately to reduce NADP^+^ to NADPH. To achieve this function, cyanobacterial PSI binds more than 100 pigments (chlorophyll and carotenoid molecules)^11^. The antenna system of cyanobacterial PSI consists of 90 Chl *a* molecules and 22 carotenes, whose function is to capture light and transfer the excitation energy to an electron transfer chain (ETC) at the center of PSI. When a pigment molecule absorbs light energy, it is absorbing photons. Each molecule can absorb a photon and transfer the resulting excitation energy through several pigments to elevate the energy level of a single electron in a special chlorophyll pair called P_700_^43^ in the photochemical reaction center. The quantum efficiency of the excitation energy transfer is very high. After excitation of any of the antenna chlorophylls the chance that the energy is successfully transferred to P_700_ and subsequent charge separation occurs is 99.98% at room temperature^46^. Charge separation and subsequent electron-transfer reactions are performed by the ETC, where energy from light excites an electron at the P_700_ site, which then proceeds down in energy through an ETC to the terminal iron-sulfur complex F_B_^47^. This process has an unprecedented quantum yield of nearly 1.0^13^. The internal quantum efficiency of nearly 100% makes PSI the most efficient energy converter in nature.

For LHCII, chlorophylls in each monomeric subunit form several clusters of strongly excitonically coupled pigments, and intra-monomer excitation energy transfer rates are very fast^48^,^49^. For LHCII trimer and its aggregate, there is a high efficiency of energy transfer from Chl *b* to Chl *a*, but the efficiency of carotenenoid-to-Chl *a* energy transfer remains constant at ~70%^50^. Recently, a chlorophyll-chlorophyll charge transfer state between the neighboring LHCII trimers has been proposed for effectively coupling with the TiO_2_ surface and thus injecting electrons into the TiO_2_ conduction band^35^. It is reasonable to deduce that a charge transfer state might also exist between the three monomers in a LHCII timer.

For 6 type bio-dye sensitized solar cell, PSI is only a shallow surface adsorption with low adsorbance (Figure 5c), but LHCII is a deep-layer adsorption with high adsorbance (Figure 5f). However, for the cell performance, both the J_SC_ and η of PSI-SSC are larger than those of LHCII-SSC (Figure 6), which verifies that PSI is characterized with efficient energy transfer in the antenna system, charge separation and transfer in the ETC, and electron injection from F_B_ to the conducting band of TiO_2_ film. Simultaneously, the oxidized P_700_, located at the lumenal side, undergoes the charge regeneration by the redox couple more easily (Figure 8a), showing high efficiency of charge regeneration. For LHCII, the efficiencies of energy transfer, charge separation and transfer, and electron injection from the charge transfer state of chlorophyll clusters to the conducting band of TiO_2_ film, are all lower. At the same time, the oxidized chlorophyll cluster, located at the stromal side, the charge regeneration by the redox couple is much harder (Figure 8b), indicating a low efficiency of charge regeneration.

For the 6+1 type bio-dye sensitized solar cell, there is a deep-layer adsorption for both PSI and LHCII in the scattering layer. In addition, in the transparent layer, PSI is only a shallow surface adsorption (Figure 5d), but LHCII is deep-layer adsorption (Figure 5g). The scattering effect significantly enhances the absorption of photons by LHCII in the transparent layer. Thus, both the J_SC_ and η of LHCII-SSC are larger than those of PSI-SSC (Figure 6).

For the 1+1 type bio-dye sensitized solar cell, similarly to the 6+1 type bio-dye SSC, there is deep-layer adsorption for both PSI and LHCII in the scattering layer. Furthermore, PSI is only a shallow surface adsorption (Figure 5e), but LHCII is a deep-layer adsorption in the 2 μm transparent layer (Figure 5h). The J_SC_ and η of PSI-SSC are therefore much larger than those of LHCII-SSC (Figure 6), which contributes to the high efficiency of PSI for energy transfer, charge separation and transfer, electron injection to the conducting band of TiO_2_ film, and charge regeneration by the redox couple (Figure 8a).

## Discussion

Three types of TiO_2_ films were designed, and bio-dyes, *A.* *platensis* PSI and spinach LHCII were spontaneously sensitized on the designed TiO_2_ electrodes, to assess the effects of pigment-protein complex on the performance of bio-solar cells. This systematic study helps to unravel the optimal pore size and thickness of TiO_2_ film, and the advantages of PSI over LHCII.

The adsorbances of PSI and LHCII on the TiO_2_ electrodes were measured by fluorescence spectrometry. Aadsorption models of bio-macromolecular PSI and LHCII on the designed TiO_2_ electrodes basing on the 3D structures of PSI and LHCII trimer, and the size of particles and inner pores in the TiO_2_ film, are proposed. The morphology of TiO_2_ film has a large effect on both the adsorbance and the depth of bio-dye adsorption. PSI can be adsorbed completely in the inner porous structure of the 2 μm scattering layer of 200 nm TiO_2_ particles, but only exhibits a shallow surface adsorption on the transparent layer of 20 nm TiO_2_ particles. LHCII can be adsorbed both in the large pore of the scattering layer and in the mesopore of the transparent layer, and the adsorption depth is larger than 2 μm in the transparent layer of the TiO_2_ film.

PSI trimer is less stable than LHCII trimer in DDM solublized solution, and some PSI trimer is disassembled to monomers. But PSI shows its merit of high efficiency for captured energy transfer in the antenna system, charge separation and transfer in ETC, and electron injection from F_B_ to the TiO_2_ conducting band. After optimization, the best performance of PSI-SSC has been enhanced from J_SC_ = 0.36 mA cm^-2^ and η = 0.08%^29^ to J_SC_ = 1.31 mA cm^−2^ and η = 0.47%; and the best performance of LHCII-SSC has been enhanced from J_SC_ = 0.80 mA cm^−2^ and η = 0.27%^34^ to J_SC_ = 1.51 mA cm^−2^ and η = 0.52%. The J_SC_ of these SSCs may have huge potential to improve, for example, by changing the redox couples in the electrolytes^51^.

Generally, a monolayer adsorption of dye molecules is optimal for DSSC functionality, since the multilayered adsorption of dye molecules is ineffective for electron injection although it may increase the optical absorption of the photoelectrode film. In this work, surfactant DDM keeps PSI suspended in solution, and forms a monolayer on the surface of TiO_2_ particles, and the V_OC_ is only 0.59 V for these designed PSI-SSCs. Recently a proof-of-concept study was report for a full solid state biophotovoltaic cell containing a PSI monolayer with the best V_OC_ 0.39 V and J_SC_ 0.31 mA cm^−2^ 52. The V_OC_ of PSI monolayer SSCs may become difficult to increase. However, large photovoltages well above 10 V can be generated by plant PSI crystal under illumination of 100 mW cm^−2^ 53, where orienting PSI individuals are properly aligned. In addition, PSI crystals are expected to be stable for years under multiple light-induced photovoltage cycles. The ability to orient PSI in films, and not just as single crystals, with inexpensive and rapid processing could lead to major advances in the performance of PSI-hybrid electrodes^18^.

## Methods

### Isolation of PSI and LHCII

The cyanobacterium *A. platensis* and spinach were used for PSI and LHCII preparation respectively. The thylakoid membranes of *A. platensis* were prepared as described previously^54^ and PSI was prepared from the TX-solubilized thylakoid membranes^42^. LHCII was isolated from fresh spinach leaves with good turgor and dark green color, according to the protocol described previously^32^,^55^.

### Fabrication of electrodes

Designed TiO_2_ electrodes were fabricated by HeptaChroma SolarTech. TiO_2_ pastes (DHS-TPP3 or DHS-TPP200) were printed on the conductive fluorine-doped tin oxide (FTO) glass (13 Ω/sq, Nippon Sheet Glass, Japan) with a layer-by-layer screen printing method^39^. The electrodes coated with TiO_2_ pastes were sintered at 500°C for 30 min. The resulting TiO_2_ films were ~2 μm thick for each layer. The counter electrodes were prepared according to the method^56^ that Pt paste (DHS-PtSP) was spread on FTO glass and heated at 450 °C for 30 min.

### Preparation of dye solutions and sensitization of TiO_2_ electrodes

Spinach LHCII, 80 μg Chl mL^−1^, was solubilized by 1.0 mM DDM in 20 mM 2-(N-morpholino) ethanesulfonic acid (MES) buffer at pH = 6.0 with 8 mM sucrose. *A.* *platensis* PSI, 80 μg Chl mL^−1^, was solubilized by 1.0 mM DDM in 20 mM MES buffer at pH = 6.5. TiO_2_ electrodes pretreated at 350 °C for 40 min were cooled down to ~50 °C and soaked into the above solutions, sealed and kept in dark at room temperature for 96 hours. Bio-electrodes were dried at 25 °C for 40 min in a vacuum drier.

### Measurement of absorption and fluorescence spectra

Steady-state UV/Vis absorption spectra were scanned at 0.5 nm resolution by a Shimadzu (Kyoto, Japan) UV/Vis 2450 spectrometer at room temperature. Steady-state fluorescence emission spectra were recorded by a FluoroMax-4 fluorescence spectrometer (Horiba Jobin Yvon) from 600 nm to 800 nm at room temperature excited at 436 nm. The dried TiO_2_ electrode was fixed on the solid sample holder at 30° angle excitation with a 515 nm cutoff filter. Total internal reflection fluorescence (TIRF) spectra were measured with an accessory TA1004, which allows for detecting molecules at the TIRF surface in the presence of relatively high concentration of the same molecules in the bulk solution.

### Adsorbance determination

The adsorbance of LHCII and PSI on TiO_2_ electrodes was determined by fluorescence spectrometry. Firstly, pigments were extracted by 80% acetone and then diluted to OD_436_ < 0.05. Fluorescence emission intensity was measured in a 5 mm × 5 mm cell with 436 nm as the excitation wavelength, and slit width set to 3 nm.

### Fabrication of DSSC

The TiO_2_ electrode sensitized with LHCII or PSI was used as the working electrode with the Pt-coated conducting glass as the counter electrode. The two electrodes were placed on top of each other using a hot-melt sealing foil of Surlyn polymer (25 µm, Solaronix SA) as a spacer to form the electrolyte space. A thin layer of electrolyte was introduced into the inter-electrode space. The electrolyte consisted of 0.5 M LiI, 0.05 M I_2_, 0.3 M DMPII, 0.5 M 4-TBP and 0.1 M GNCS in acetonitrile. The assembled DSSC was attached to an optical shielding mask to confine the effective irradiation area to 0.25 cm^2^.

### Characterization of DSSC

The current-voltage characteristics of each cell were recorded with a Keithley 2400 sourcemeter using a 300 W xenon light source (Newport Oriel 91160-1000) equipped with AM 1.5 filter, which was focused to give a light intensity 100 mW/cm^2^, on the surface of dye-sensitized TiO_2_ electrodes. In order to reduce scattered light from the edge of the TiO_2_ electrodes, a light shading mask was used to cover the DSSC to fix an active area of 0.25 cm^2^. The current output of each cell was recorded by linearly varying the potential from −0.1 to 0.8 V in a 4-wire configuration. The incident photo-to-current conversion efficiency (IPCE) was measured as a function of excitation wavelength using the incident light from an Osram 150 W tungsten halogen lamp (CrownTech CTTH-150W), which was focused through a 1/4 m monochromator (Spectral Product DK240) on the DSSC under test.

## Author Contributions

D.Y., B.G. and F.H. conceived the idea and designed the experiments. D.Y., M.W., G.Z. and S.L. performed the experiments. D.Y., B.G., S.L. and F.H. discussed and wrote the paper.

## Supplementary Material

Supplementary Information Supplementary information

## Figures and Tables

**Table 1:** Adsorbance of PSI and LHCII on TiO_2_ electrodes

Bio-dye	Type of TiO_2_ electrode	Adsorbance (μg Chl cm^−2^)
*A.* *platensis* PSI	6	1.39 ± 0.09
	6+1	2.70 ± 0.34
	1+1	2.84 ± 0.12
Spinach LHCII	6	4.72 ± 0.20
	6+1	6.00 ± 0.20
	1+1	5.22 ± 0.04
