# Supplementary material for: Enhanced photocurrent production by bio-dyes of photosynthetic macromolecules on designed TiO2 film
Source: Sci Rep. 2015 Mar 20;5:9375. doi: 10.1038/srep09375 (PMC4366820; doi:10.1038/srep09375)
Supplement: Supplementary Information [file srep09375-s1.pdf]

Supplementary Information

Enhanced photocurrent production by bio-dyes of photosynthetic macromolecules on designed TiO_2 film

Daoyong Yu[1,2,*], Mengfei Wang[2], Guoliang Zhu[2], Baosheng Ge[1,2], Shuang Liu[2], Fang Huang[1,2,*]

[1]*State Key Laboratory of Heavy Oil Processing, China University of Petroleum (East China), Qingdao 266580, China*
[2]*Center for Bioengineering and Biotechnology, China University of Petroleum (East China), Qingdao 266580, China*

*Corresponding authors at: Center for Bioengineering and Biotechnology
China University of Petroleum (East China)
66 West Changjiang Road, Qingdao 266580, China

E-mail address: daoyong@upc.edu.cn (D. Yu)
fhuang@upc.edu.cn (F. Huang)

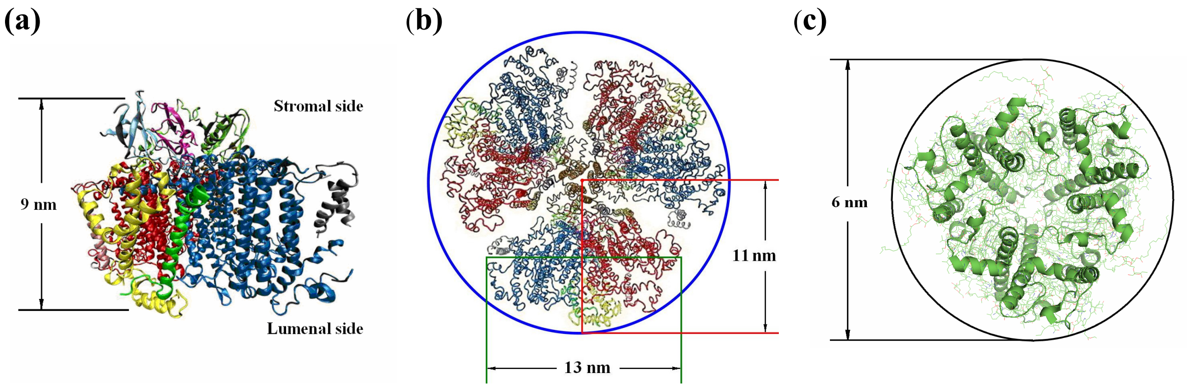

Figure S1 Dimensional size of PSI (PDB ID: 1JB0) and LHCII (PDB ID: 1RWT). (**a**) PSI monomer. (**b**) Lumenal side of trimeric PSI complex constructed from 1JB0. (**c**) LHCII trimer. Marked sizes are measured by PyMOL.

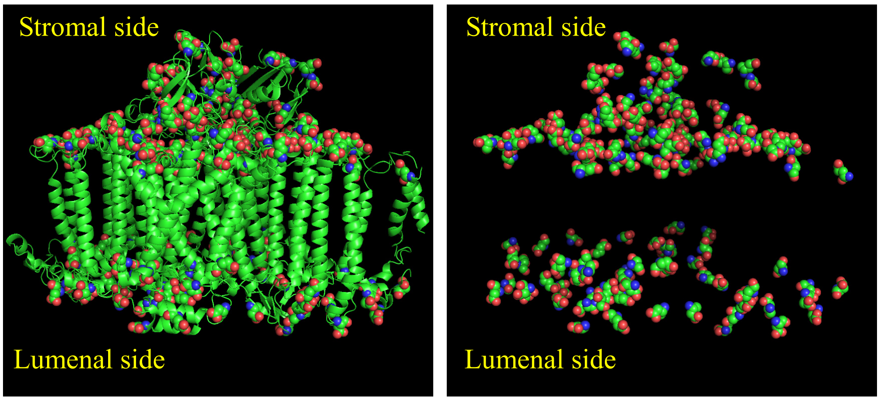

(**a**) Distribution of residues of aspartic acid and glutamic acid (Lateral view)

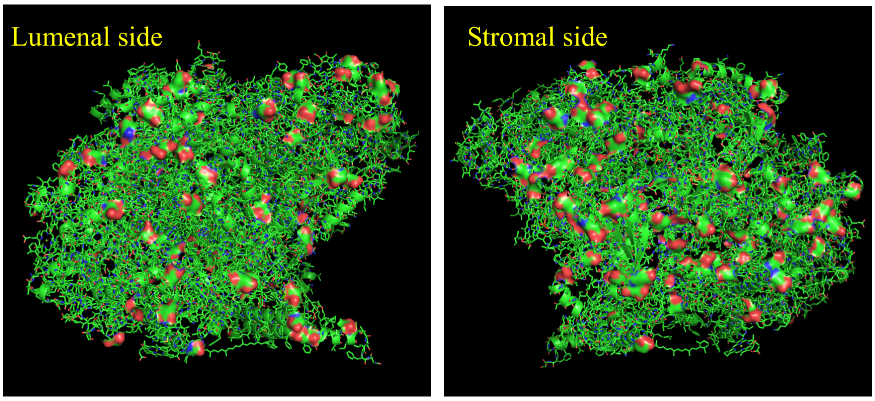

Bottom view Top view

(**b**) Negative charged surface of residues of aspartic acid and glutamic acid

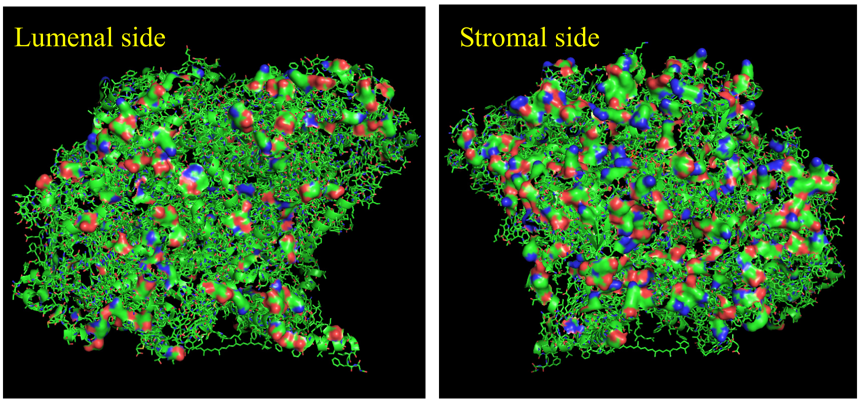

Bottom view Top view

(**c**) Charged surface of negative residues of D and E, and positive residues of H, R, and K

Figure S2 PSI monomer, Deduced from PDB ID 1JB0. (**a**) There are 102 carboxyl residues (55E+47D) at the stromal side, and there are 58 carboxyl residues (15E+43D) at the lumenal side (PDBTM 1jb0). (**b**) The stromal side has more carboxyl groups on the surface. (**c**) The stromal side has more charged groups of carboxyl and amino on the surface.

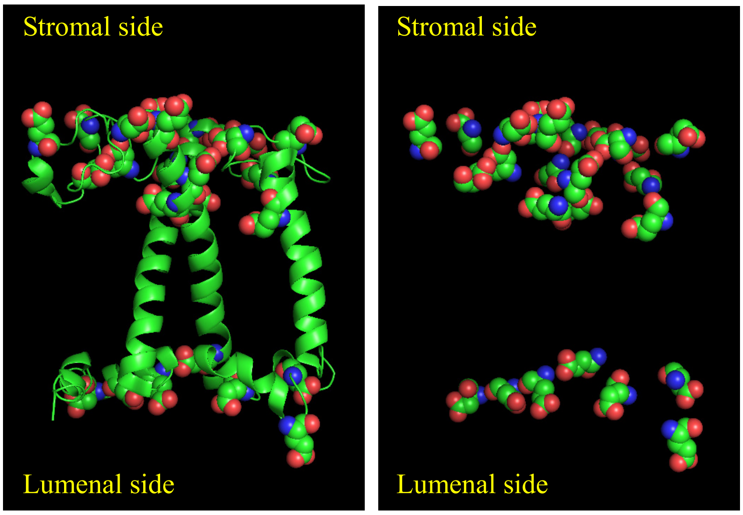

(**a**) Distribution of residues of aspartic acid and glutamic acid (Lateral view)

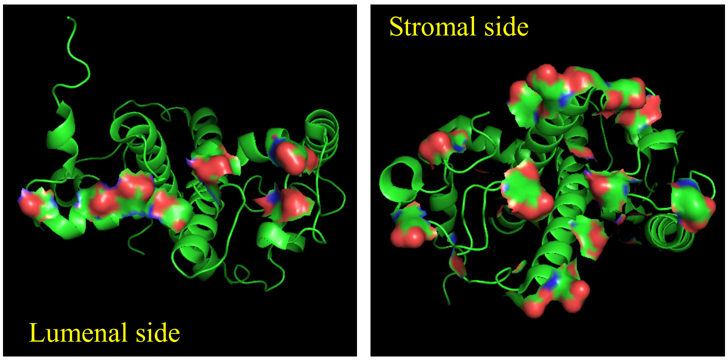

Bottom view　　　Top view

(**b**) Negative charged surface of residues of aspartic acid and glutamic acid

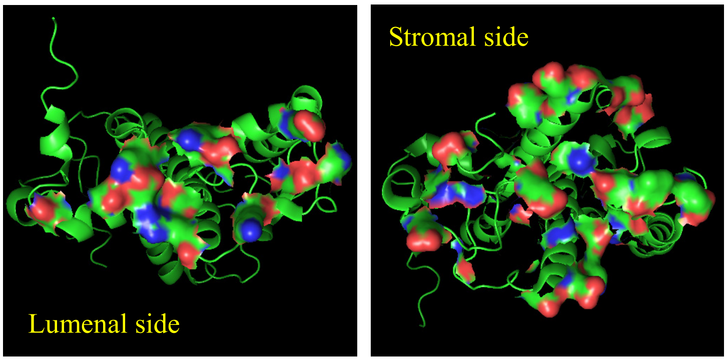

Bottom view　　　Top view

(**c**) Charged surface of negative residues of D and E, and positive residues of H, R, and K

Figure S3　LHCII monomer, Deduced from PDB ID 1RWT. (**a**) There are 18 carboxyl residues (10E+8D) at the stromal side, and there are only 7 carboxyl residues (4E+3D) at the lumenal side. (**b**) The stromal side has more carboxyl groups on the surface. (**c**) The stromal side has more charged groups of carboxyl and amino on the surface.

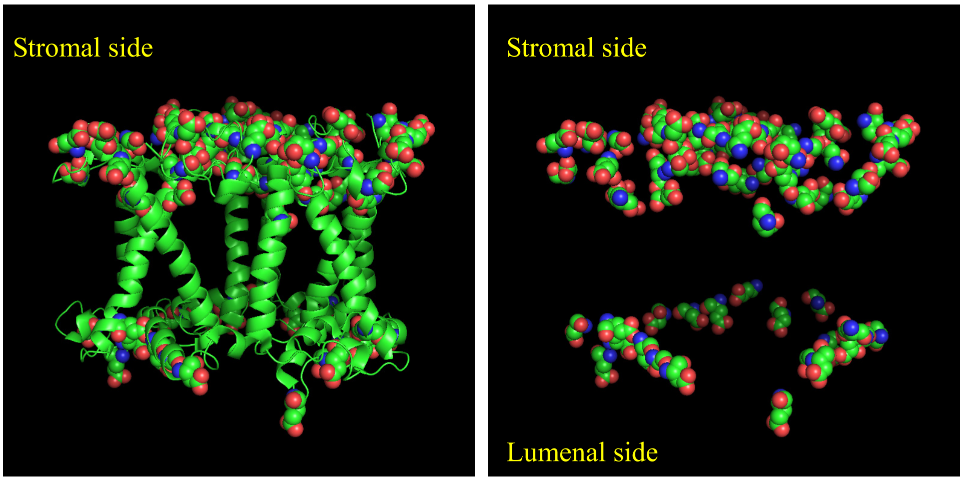

(**a**) Distribution of residues of aspartic acid and glutamic acid (Lateral view)

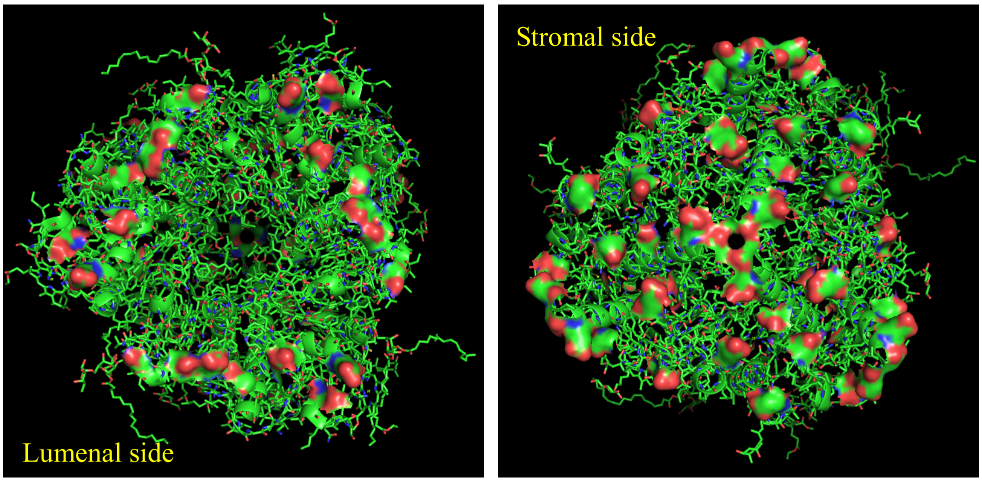

Bottom view Top view

(**b**) Negative charged surface of residues of aspartic acid and glutamic acid

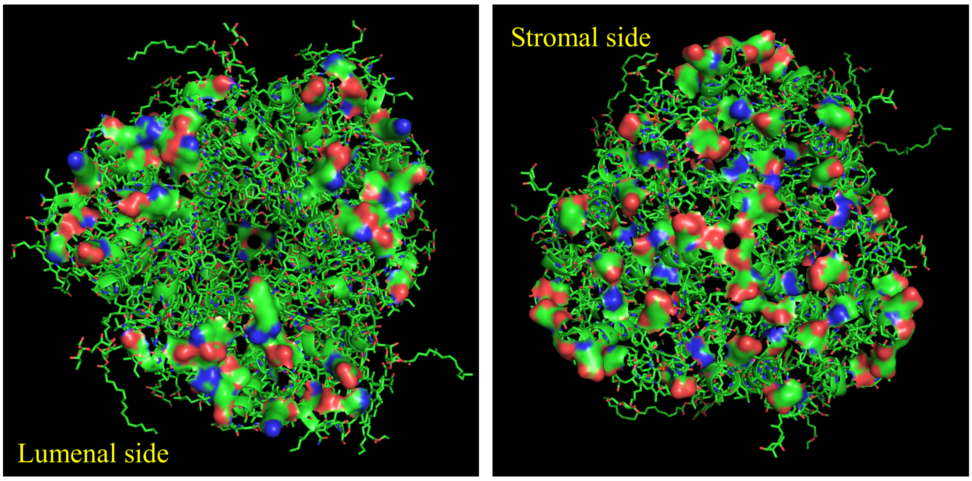

Bottom view Top view

(**c**) Charged surface of negative residues of D and E, and positive residues of H, R, and K

Figure S4 LHCII trimer, Deduced from PDB ID 1RWT. (**a**) There are 54 carboxyl residues (30E+24D) at the stromal side, and there are only 21 carboxyl residues (12E+9D) at the lumenal side. (**b**) The stromal side has more carboxyl groups on the surface. (**c**) The stromal side has more charged groups of carboxyl and amino on the surface.

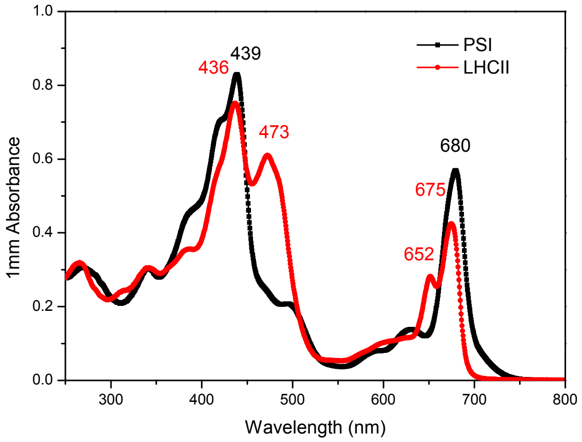

Figure S5 Absorption spectra of *A. platensis* PSI trimer and spinach LHCII trimer in solutions. Spinach LHCII, 80μg Chl/mL, was solubilized by 1.0 mM DDM in 20 mM MES buffer at pH=6.0 with 8 mM sucrose. *A. platensis* PSI, 80μg Chl/mL, was solubilized by 1.0 mM DDM in 20 mM MES buffer at pH=6.5. Steady-state UV/Vis absorption spectra were scanned at 0.5 nm resolution by a Shimadzu (Kyoto, Japan) UV/Vis 2450 spectrometer at room temperature. The maxima of LHCII in the Qy region are around 675 nm and 652 nm, and in the Soret region around 436 nm and 473 nm. The maxima of PSI in the Qy region and Soret region are at about 680 nm and 439 nm respectively, and these are characteristic peaks of Chl *a* assembled in PSI complexes, while the shoulder peak at about 495 nm is contributed by carotenoids assembled in PSI complexes.

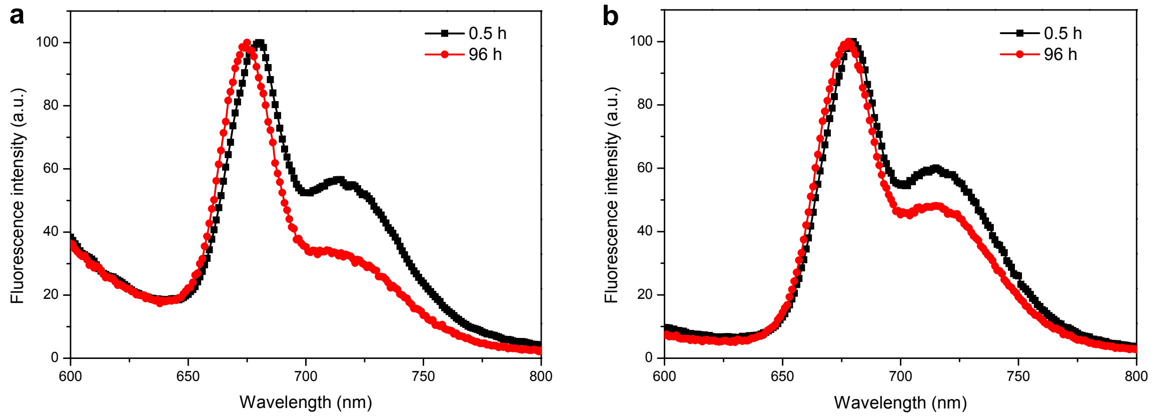

Figure S6 TIRF spectra of *A. Platensis* PSI solubilized by 1.0 mM DDM in 20 mM MES buffer at pH 6.5. (**a**) PSI, 80 μg Chl/mL. (**b**) PSI, 400 μg Chl/mL. TIRF spectra were measured with an accessory TA1004 on a FluoroMax-4 fluorescence spectrometer (Horiba Jobin Yvon) from 600 nm to 800 nm at room temperature excited at 436 nm, and slit width of excitation and emission were set at 5 nm and 5 nm respectively. The TA1004 accessory allows for detecting molecules at the TIRF surface in the presence of relatively high concentration of the same molecules in the bulk solution.

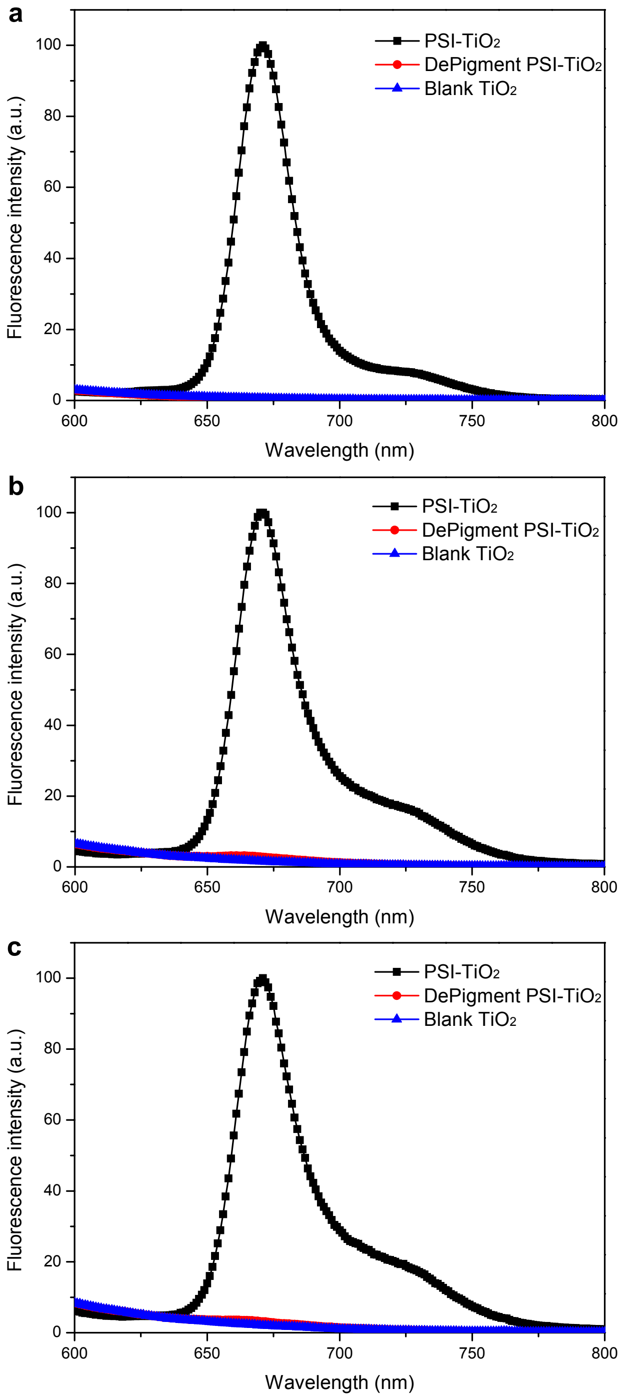

Figure S7 Fluorescence spectra of PSI-TiO_2 electrodes before and after extraction of pigments by 80% acetone/water. (**a**) 6 type, (**b**) 6+1 type, and (**c**) 1+1 type TiO_2 electrodes.

Fluorescence emission spectra were recorded by a FluoroMax-4 fluorescence spectrometer (Horiba Jobin Yvon) from 600 nm to 800 nm at room temperature excited at 436 nm, and slit width of excitation and emission were set at 3 nm and 1 nm respectively. Dried TiO_2 electrode was fixed onto the solid sample holder at 30° angle excitation with a 515 nm cutoff filter.

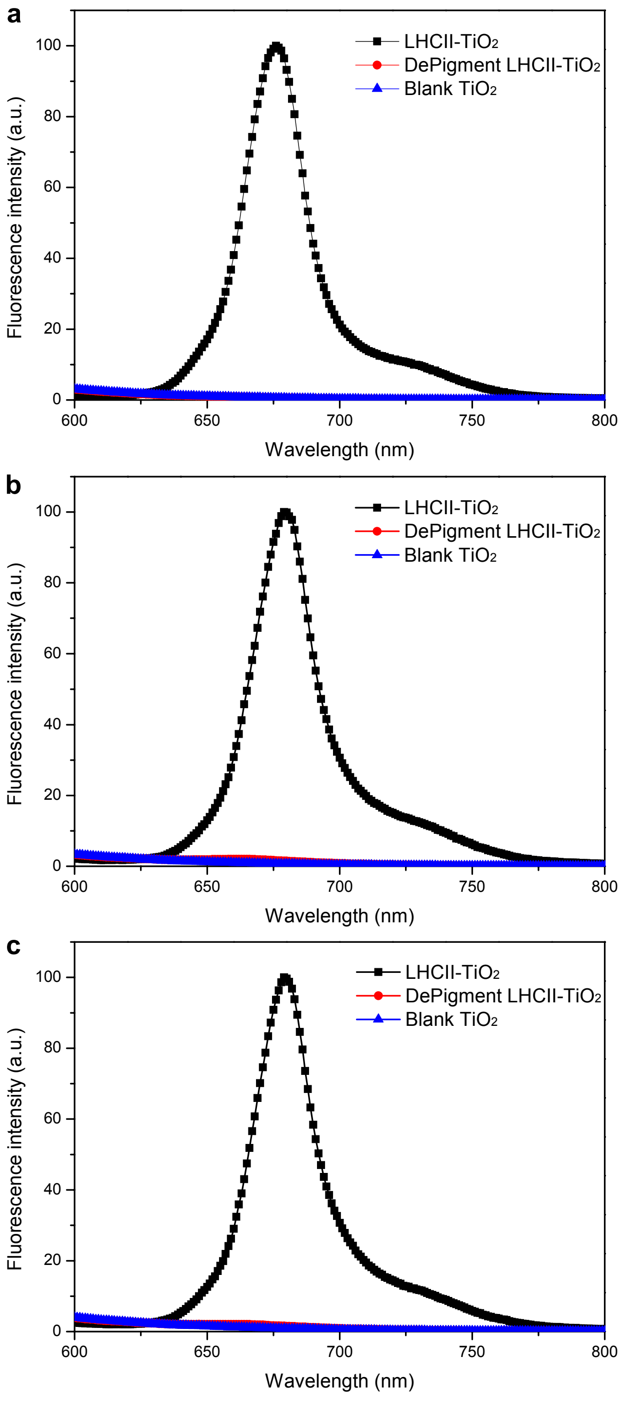

Figure S8 Fluorescence spectra of LHCII-TiO_2 electrodes before and after extraction of pigments by 80% acetone/water. (**a**) 6 type, (**b**) 6+1 type, and (**c**) 1+1 type TiO_2 electrodes.

Fluorescence emission spectra were recorded by a FluoroMax-4 fluorescence spectrometer (Horiba Jobin Yvon) from 600 nm to 800 nm at room temperature excited at 436 nm, and slit width of excitation and emission were set at 3 nm and 1 nm respectively. Dried TiO_2 electrode was fixed onto the solid sample holder at 30° angle excitation with a 515 nm cutoff filter.

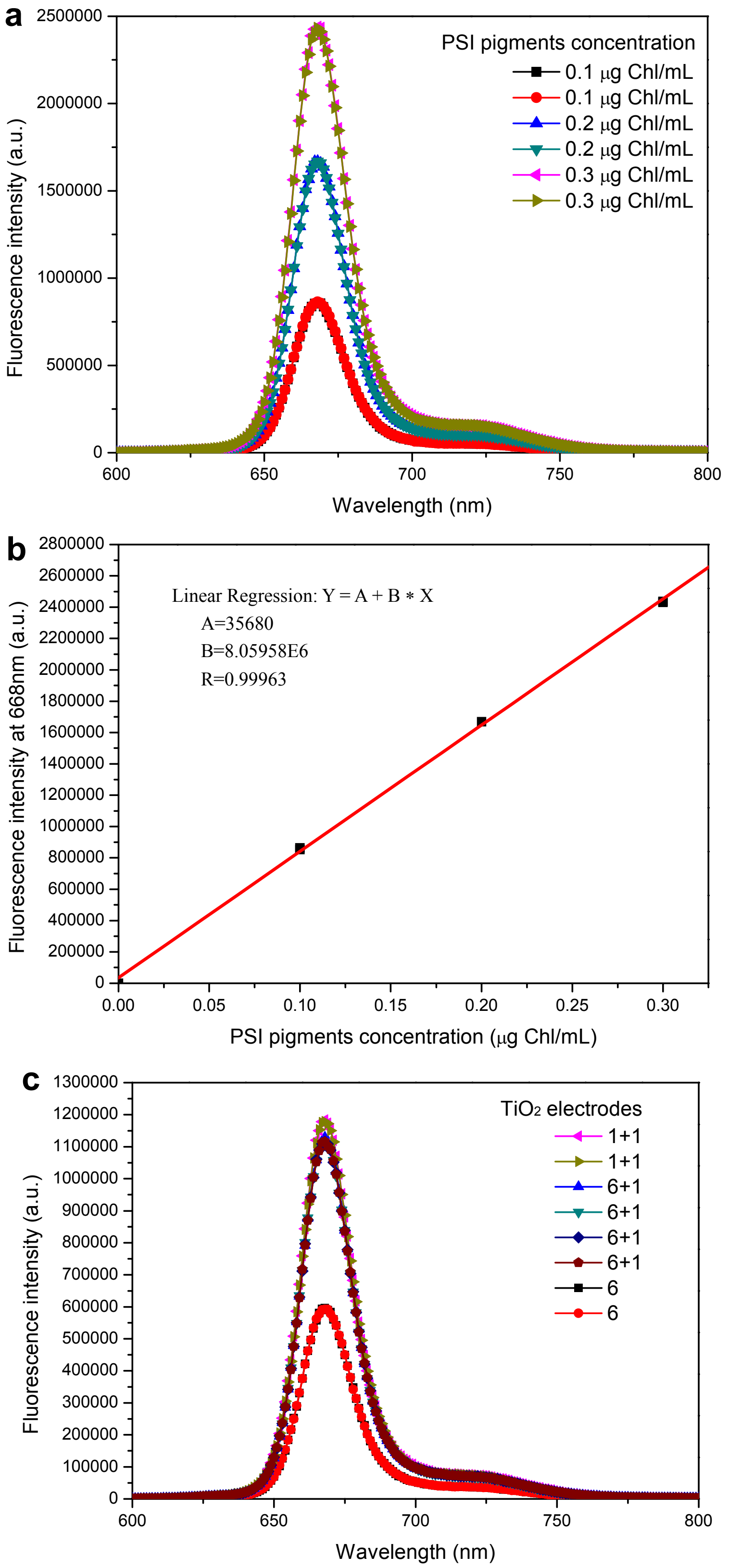

Figure S9 Adsorbance determination of PSI on TiO_2 electrodes. (**a**) Fluorescence spectra of standard solutions of PSI pigments in 80% acetone/water. (**b**) The linear relationship of fluorescence intensity at 668 nm with concentration of PSI pigments. (**c**) Fluorescence spectra of extracted pigments from PSI-TiO_2 electrodes for quantitative analysis.

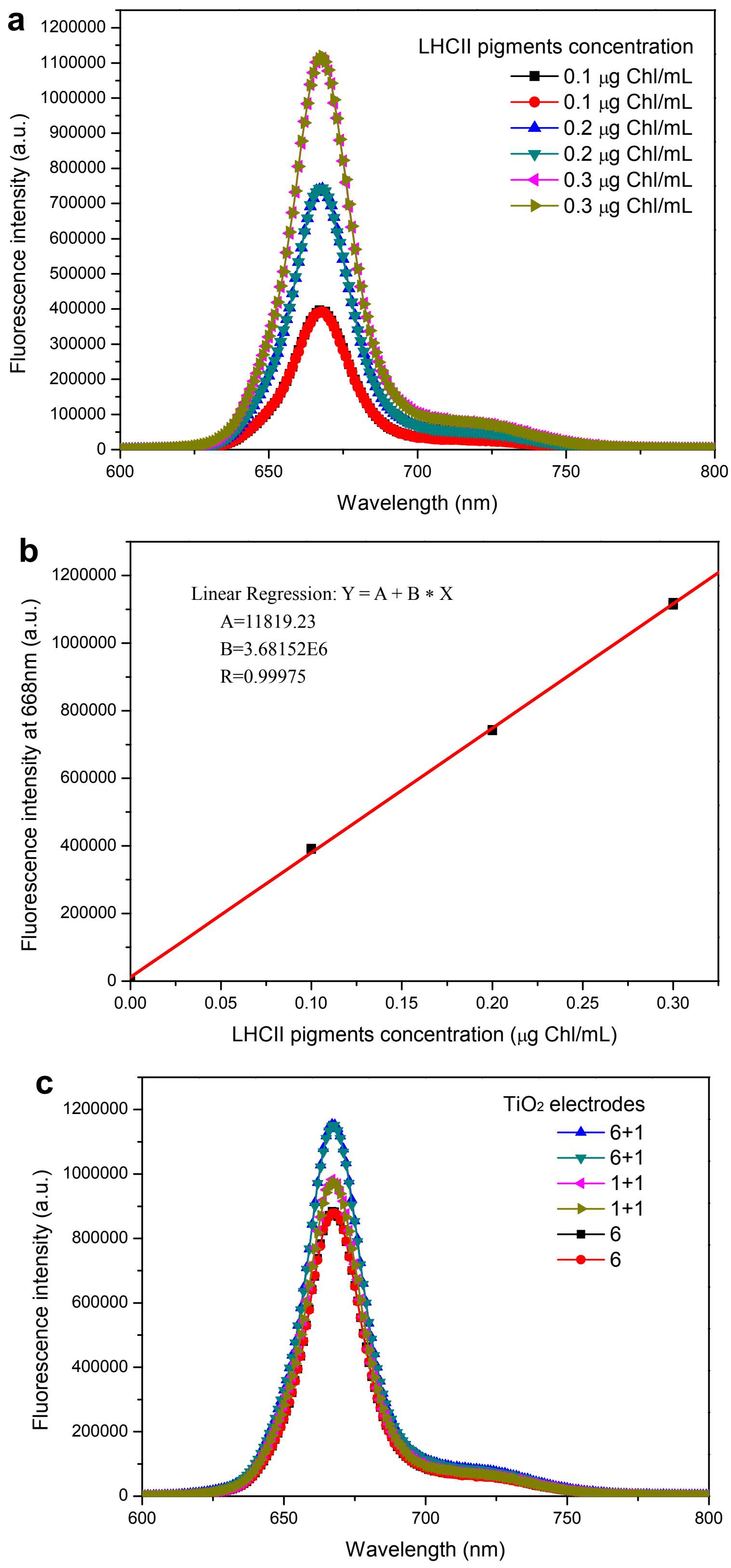

Figure S10 Adsorbance determination of LHCII on TiO_2 electrodes. (**a**) Fluorescence spectra of standard solutions of LHCII pigments in 80% acetone/water. (**b**) The linear relationship of fluorescence intensity at 668 nm with concentration of LHCII pigments. (**c**) Fluorescence spectra of extracted pigments from LHCII-TiO_2 electrodes for quantitative analysis.

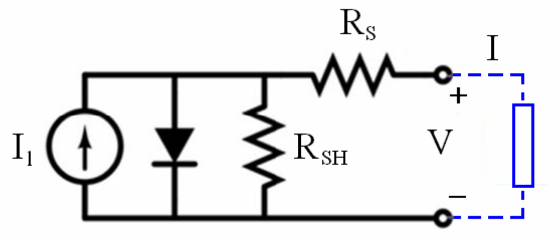

Figure S11 Simplified equivalent circuit model for a solar cell. R_S and R_{SH} represent the series and shunt resistance. http://www.ni.com/white-paper/7230/en/
